# Recommendations for performance optimizations when using GATK3.8 and GATK4

**DOI:** 10.1186/s12859-019-3169-7

**Published:** 2019-11-08

**Authors:** Jacob R Heldenbrand, Saurabh Baheti, Matthew A Bockol, Travis M Drucker, Steven N Hart, Matthew E Hudson, Ravishankar K Iyer, Michael T Kalmbach, Katherine I Kendig, Eric W Klee, Nathan R Mattson, Eric D Wieben, Mathieu Wiepert, Derek E Wildman, Liudmila S Mainzer

**Affiliations:** 1 National Center for Supercomputing Applications, University of Illinois at Urbana-Champaign, 1205 W. Clark St., Urbana, IL, USA; 2 Mayo Clinic, Department of Research Services, 200 1st St. SW, Rochester, MN, USA; 3 Mayo Clinic, Department of IT Executive Administration, 200 1st St. SW, Rochester, MN, USA; 4 Mayo Clinic, Department of Health Sciences Research, 200 1st St. SW, Rochester, MN, USA; 5 Department of Crop Sciences, University of Illinois at Urbana-Champaign, 1102 S. Goodwin Ave., Urbana, IL, USA; 6 Department of Electrical and Computer Engineering, University of Illinois at Urbana-Champaign, 306 N. Wright St., Urbana, IL, USA; 7 Mayo Clinic, Department of Biochemistry and Molecular Biology, 200 1st St. SW, Rochester, MN, USA; 8 Department of Molecular and Integrative Physiology, University of Illinois at Urbana-Champaign, 407 S. Goodwin Ave., Urbana, IL, USA; 9 Institute for Genomic Biology, University of Illinois at Urbana-Champaign, 1206 W Gregory Dr., Urbana, IL, USA

**Keywords:** GATK, Genomic variant calling, Best practices, Computational performance, Cluster computing, Parallelization

## Abstract

**Background:**

Use of the Genome Analysis Toolkit (GATK) continues to be the standard practice in genomic variant calling in both research and the clinic. Recently the toolkit has been rapidly evolving. Significant computational performance improvements have been introduced in GATK3.8 through collaboration with Intel in 2017. The first release of GATK4 in early 2018 revealed rewrites in the code base, as the stepping stone toward a Spark implementation. As the software continues to be a moving target for optimal deployment in highly productive environments, we present a detailed analysis of these improvements, to help the community stay abreast with changes in performance.

**Results:**

We re-evaluated multiple options, such as threading, parallel garbage collection, I/O options and data-level parallelization. Additionally, we considered the trade-offs of using GATK3.8 and GATK4. We found optimized parameter values that reduce the time of executing the best practices variant calling procedure by 29.3% for GATK3.8 and 16.9% for GATK4. Further speedups can be accomplished by splitting data for parallel analysis, resulting in run time of only a few hours on whole human genome sequenced to the depth of 20X, for both versions of GATK. Nonetheless, GATK4 is already much more cost-effective than GATK3.8. Thanks to significant rewrites of the algorithms, the same analysis can be run largely in a single-threaded fashion, allowing users to process multiple samples on the same CPU.

**Conclusions:**

In time-sensitive situations, when a patient has a critical or rapidly developing condition, it is useful to minimize the time to process a single sample. In such cases we recommend using GATK3.8 by splitting the sample into chunks and computing across multiple nodes. The resultant walltime will be nnn.4 hours at the cost of $41.60 on 4 c5.18xlarge instances of Amazon Cloud. For cost-effectiveness of routine analyses or for large population studies, it is useful to maximize the number of samples processed per unit time. Thus we recommend GATK4, running multiple samples on one node. The total walltime will be ∼34.1 hours on 40 samples, with 1.18 samples processed per hour at the cost of $2.60 per sample on c5.18xlarge instance of Amazon Cloud.

## Background

The evolution of sequencing technologies [1, 2] encouraged many applications of Whole Genome Sequencing (WGS) and Whole Exome Sequencing (WES) in genomic research and the clinic [3, 4]. One of these applications is genomic variant calling, commonly performed using the Genome Analysis Toolkit (GATK), maintained by the Broad Institute [5–8]. As sequencing machines become faster and cheaper [9], analysis must speed up as well. Yet variant calling analysis using GATK still takes many hours, or even days, on deeply sequenced samples [10–13]. A number of proprietary solutions have emerged in response to this over the last five years, such as Isaac [14], Sentieon’s DNASeq [15, 16], Genalice [17] and Dragen [18]. However, they are either closed-source or do not follow the GATK Best Practices [7, 8]. Accelerating the GATK open-source code itself is of tremendous interest to the bioinformatics community, for the sake of reproducibility and openness of biomedical research. To this end the Broad Institute partnered with Intel to introduce computational performance optimizations [19–21]. GATK3.8 is the latest release of the "traditional" Java-based GATK designed to work on regular servers or compute clusters, and was announced to contain significant computational performance improvements through the collaboration with Intel [22].

In addition to optimizations of the traditional variant calling algorithms [10–13], the community also has been calling for a variant calling toolkit that can take advantage of dedicated MapReduce platforms, as Hadoop [23] and especially Spark [24–26] are more appropriate for this type of genomic data analysis compared to traditional high performance computing (HPC). Thus GATK4, first officially released in January of 2018, is meant to be eventually deployed on data analytics platforms. At present it contains both Spark and non-Spark implementations of many of the tools and is thus still runnable in traditional HPC clusters. Yet even the non-Spark implementation has been significantly rewritten relatively to the GATK3.x versions, to improve maintainability and speed.

How do these changes affect the deployment practices of GATK-based variant calling workflows in production clinical and research settings, and what are the optimal patterns of deployment? We are the first to have performed a detailed scalability analysis of these new GATK versions to ascertain the advertised speedup. Based on those results we have developed appropriate sample-based parallelization techniques and deployment recommendations for the end users. Because most of the Spark tools were still in beta at the time of the initial release, we focused our testing on the non-Spark implementations.

When optimizing a workflow, one can perform two distinct optimizations, and we explore them both:

**maximizing speed** to minimize the time to process a single sample; useful in time-critical situations, i.e. when a patient has a critical or rapidly developing condition;

**maximizing throughput** to maximize the number of samples processed per unit time; cost-effective for routine analyses or large population studies.

Overall we did find that both GATK versions yield an impressive walltime <4 hours (excluding alignment) on a 20X WGS human data, with appropriate sample-level parallelization.

## Implementation

We implemented a battery of benchmarkingscripts to perform the testing of GATK3.8 and GATK4 tools, as described below.

### Software versions

GATK3.8 was downloaded from the Broad Institute’s softwaredownloadpage , build GATK-3.8-0-ge9d806836. Picard version 2.17.4 and GATK4.0.1.2 were downloaded from GitHub as pre-compiled jar files.

### Tools

Our benchmarking focused on the GATK Best Practices [7, 8] starting from the duplicate marking stage through variant calling. The MarkDuplicates tool is not part of GATK3 and was called from a separate toolkit, Picard. MarkDuplicates is included directly into GATK4. Realignment is no longer recommended, and was not tested. The base recalibration process consists of two tools, BaseRecalibrator and PrintReads(GATK3.8)/ApplyBQSR(GATK4). The final tool we benchmarked was HaplotypeCaller, which is common to both versions of GATK.

### Data

A dataset corresponding to whole genome sequencing (WGS) performed on NA12878 [27, 28] to ∼20X depth was downloaded from Illumina BaseSpace on Dec 16, 2016. The paired-ended, 126 nt reads were aligned with BWA MEM [29] against the hg38 human reference (Oct 2017 GATK Bundle) and sorted with Novosort [30] prior to benchmarking. Some settings required multiple tests and measurements; in those cases we only used the reads that mapped to chromosome 21. For known sites, dbSNP build 146 was used.

### Hardware

All tests were conducted on Skylake Xeon Gold 6148 processors with 40 cores, 2.40 GHz. Each node had 192 GB, 2666 MHz RAM. The nodes were stateless, connected to a network-attached IBM GPFS ver. 4.2.1 with custom metadata acceleration. The cluster used EDR InfiniBand with 100 Gb/sec bandwidth, 100 ns latency. Nodes ran Red Hat Enterprise Linux 6.9.

## Results

### GATK3.8 tool-level thread scalability

Threading is one way of implementing parallelization to speed up a program. Data-level parallelization is frequently used in bioinformatics, by subdividing the input data into smaller chunks that can be worked on in parallel by the threads. It is useful to know how well a program scales with thread count: ideally the run time should decrease proportionately to the number of threads used on the data The non-Spark GATK4 version is entirely single-threaded, except for the PairHMM portion of HaplotypeCaller (“PairHMM scalability in GATK4 haplotypeCaller” section below). Picard’s MarkDuplicates is also single-threaded. Thus, our thread scalability testing focused on the GATK3.8 tools, which utilizes user-level options (-nct and -nt) to control how many computer cores should be engaged by the program, and how many threads one should deploy per core. We measured the walltime for each tool when invoked with a certain thread count, in the range from 1 to 40. We kept nt at 1 and modified nct, aiming to engage multiple cores on our nodes and varying the number of software threads running on the multi-core CPU. When reporting one thread for HaplotypeCaller, we mean that one thread of each type was used. We tracked the number of cores engaged and the number of threads spawned via the linux top command.

**Fig. 1:**
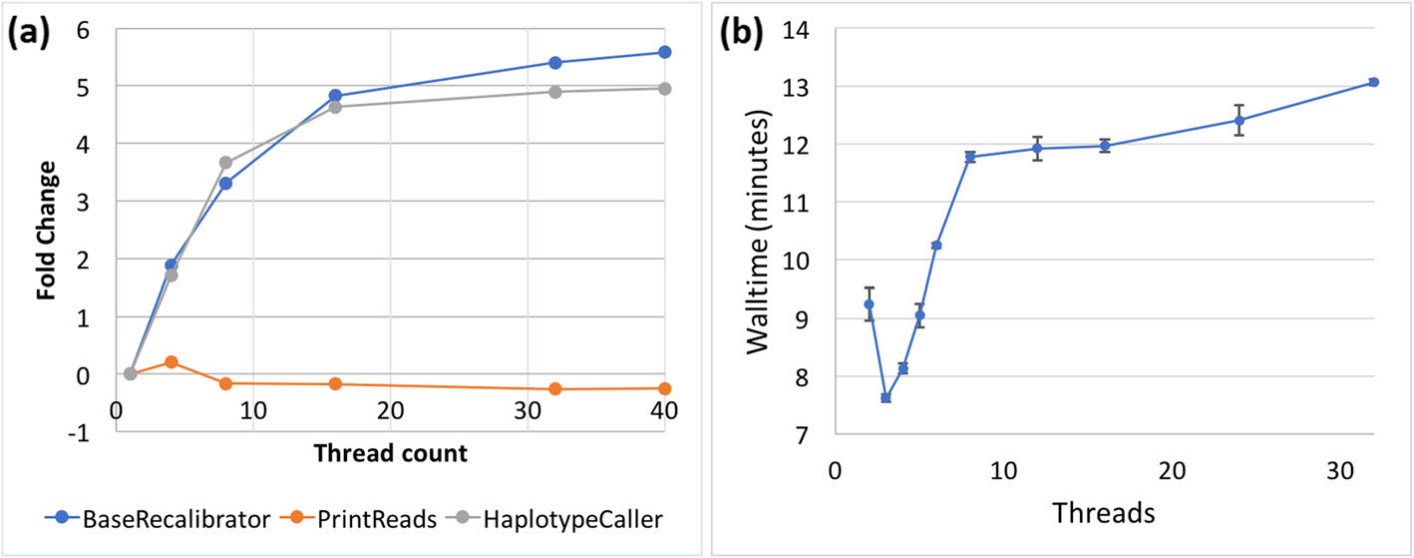
GATK3.8 Thread Scalability. **a** Scalability of BaseRecalibrator, PrintReads and HaplotypeCaller. Sample: NA12878 WGS. Fold change refers to the fold difference in walltime between the new measurement when compared to the performance with a single thread ((*newtime*−*baselinetime*)/*baselinetime*). **b** Scalability of PrintReads, in more detail. Normally walltime should decrease with thread count, as the computation is performed in parallel by multiple threads. However, in the case of PrintReads the opposite is observed. The increasing walltime as a function of thread count therefore signifies poor scalability and explains the decreasing trend for PrintReads line on panel (a). Sample: NA12878 chr 21. Error bars denote 1 SD around the mean of three replicates

The tools respond differently to multithreading, and all show suboptimal scalability: run time decreases less than the increase factor of the thread count. Both BaseRecalibrator and HaplotypeCaller experience a 5-fold speedup compared to a single-threaded run when using 16 threads, but do not scale beyond that (Fig. 1a). PrintReads gains an initial improvement with 3 threads (the apparent optimum for our dataset), and experiences degraded performance at higher thread counts (Fig. 1b).

Suboptimal scalability can occur for a variety of reasons. In the I/O-heavy bioinformatics applications, which frequently have to repeatedly grab data from disk, do work in RAM, then write back to disk, the performance usually degrades due to disk access latency, network latency in communicating to the filesystem, and thread contention for RAM bandwidth. Thus, requesting many threads is not optimal for the GATK3.8 tools, and one has to balance the number of tools running per-node vs. the number of threads requested per-tool, to ensure full node utilization without degraded performance. Performance gains could be achieved by using internal SSDs on the compute nodes, thus avoiding the network and spinning disk access issues during the computation.

### GATK4 parallel garbage collection

Garbage Collection in JAVA is a mechanism to automatically remove from memory the variables and objects that are no longer useful or necessary for computation. This frees the developer from the need to worry about manually destroying those objects in the code, thus reducing the code base and eliminating the possibility of ”forgetting” to do this, which otherwise could result in out-of-memory errors. This is a very useful feature in JAVA, and worth paying attention to when optimizing runtime performance in GATK, which is JAVA-based code. A previous study [10] found that enabling Java parallel garbage collector (PGC) with up to 32 threads improved the walltime of GATK3.7. We explored this effect in the GATK4 tools.

The flags enabling PGC are passed to the GATK4 launch script via the “–java-options” flag:


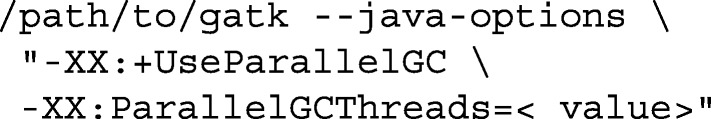


**Fig. 2:**
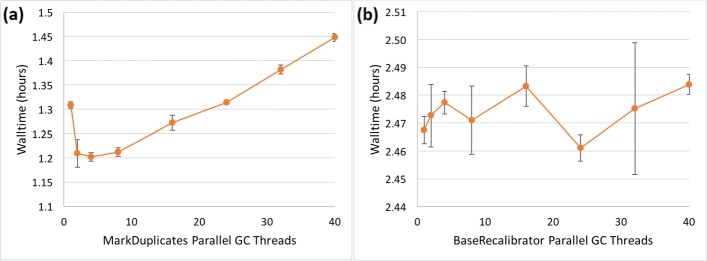
GATK4 thread scalability for Java parallel garbage collection. Sample: NA12878 WGS. The measurements at 1 PGC thread represent the default, meaning that PGC is not enabled. Error bars denote SD around the mean of three replicates. **a** MarkDuplicates. **b** BaseRecalibrator

We found that enabling PGC for either ApplyBQSR or HaplotypeCaller had no impact or even degraded performance, depending on the number of threads used (data not shown). However, in MarkDuplicates using 2-4 PGC threads provided optimal performance (Fig. 2a). For BaseRecalibrator, there is much more variability that we could not link to the state of the cluster (Fig. 2b). The optimal thread choice appears to be around 24 threads, but the high walltimes at thread counts close to 24 suggest that it may be more reliable for end users to 1) perform a similar thread count sweep on one’s own system to find the optimum, or 2) leave parallel garbage collection off to avoid one of the sub-optimal thread counts.

We took a cursory look at PGC scalability in GATK3.8 and did not find significant improvements. In Picard’s MarkDuplicates, the optimum lies at approximately 2 PGC threads.

It is not clear why GATK4 performance could not be improved by using PGC multithreading to the same extent as has been reported for GATK3.7, except that perhaps GATK4 code was still relatively fresh at the time of our testing, and further improvements would have been made later. We recommend users to run a cursory PGC thread scalability analysis on their systems to establish how GATK4 tools behave on their specific hardware. The extra human time spent doing this could buy substantial walltime and therefore financial savings, if the facility must provide high-throughput analysis of large volumes of genomic data on a continuous basis.

### Asynchronous i/O in GATK 4

GATK4 has two types of asynchronous read/write options: Samtools I/O and Tribble I/O. “Tribble” is a specialized data format, mainly used for index files. To enable asynchronous I/O, one must edit the following variables in a gatk-properties file, located at src/main/resources/org/broadinstitute/hellbender/utils/config/GATKConfig.properties in the GATK GitHub repository:


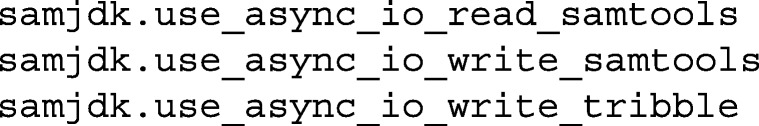


Each of these variables can be either “true” or “false”. The properties file is passed to GATK with the “–gatk-config-file” flag. Because GATK4 MarkDuplicates is just a port of Picard’s tool of the same name, it does not accept a configuration file. We ran HaplotypeCaller with a single thread for this series of tests.

We found it best to enable asynchronous I/O for Samtools reading and writing and disable it for Tribble I/O (Table 1).

**Table 1:** Effects of asynchronous I/O settings on walltime (hours) in GATK4

	Async I/O activated?		
Tool Name	no	all	only for samtools I/O
BaseRecalibrator	4.07	2.95	2.88
ApplyBQSR	2.38	2.07	2.08
HaplotypeCaller	17.25	17.31	17.08

Sample: NA12878 WGS.

### PairHMM scalability in GATK4 haplotypeCaller

Intel partnered up with the Broad Institute to create the Genomics Kernel Library (GKL), which includes key optimizations to the HaplotypeCaller algorithm. The library introduces AVX optimized versions of the PairHMM and Smith-Waterman algorithms. Additionally, OpenMP support was added to the PairHMM algorithm to enable multithreading. While the library was developed to be used in GATK4, the AVX capabilities were back propagated to GATK3.8 as well.

The pre-built GATK4 that we downloaded from the repository was already configured to automatically detect hardware support for AVX. On our Skylake architecture, AVX-512 was utilized automatically.

The multi-threaded implementation of the PairHMM algorithm can be enabled with the following flags:


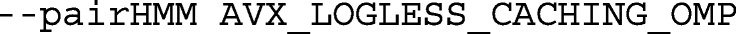


and


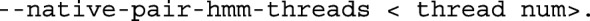


**Fig. 3:**
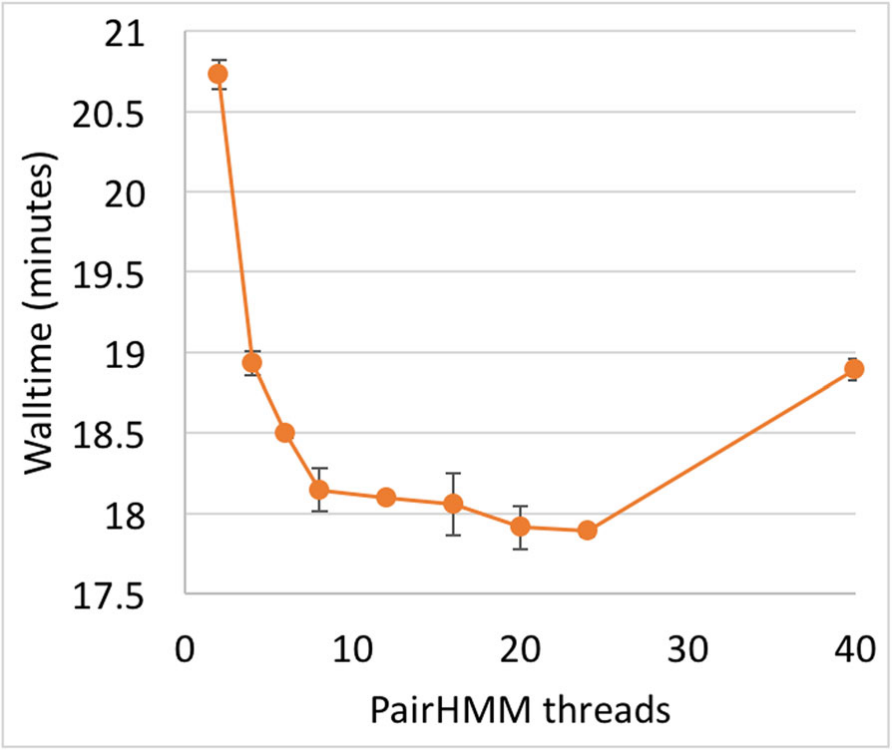
GATK4 thread scalability in HaplotypeCaller. Sample: NA12878 chr21. Error bars denote 1 SD around the mean of three replicates

The optimum for GATK4 HaplotypeCaller seems to be around 10 threads (Fig. 3).

### Splitting by chromosome

To achieve the greatest speedup, it is often efficient to split data by chromosome and process each interval in parallel. Here, we split the aligned sorted BAM into varying numbers of roughly equal-size chunks (Table 2) by using the GATK interval flag (-L) to observe how splitting affected walltime. The chunks were either kept on the same node for maximal utilization of cores (“within-node” parallelization) or spilled to more nodes for even shorter walltime (“across-node” parallelization).

**Table 2:**
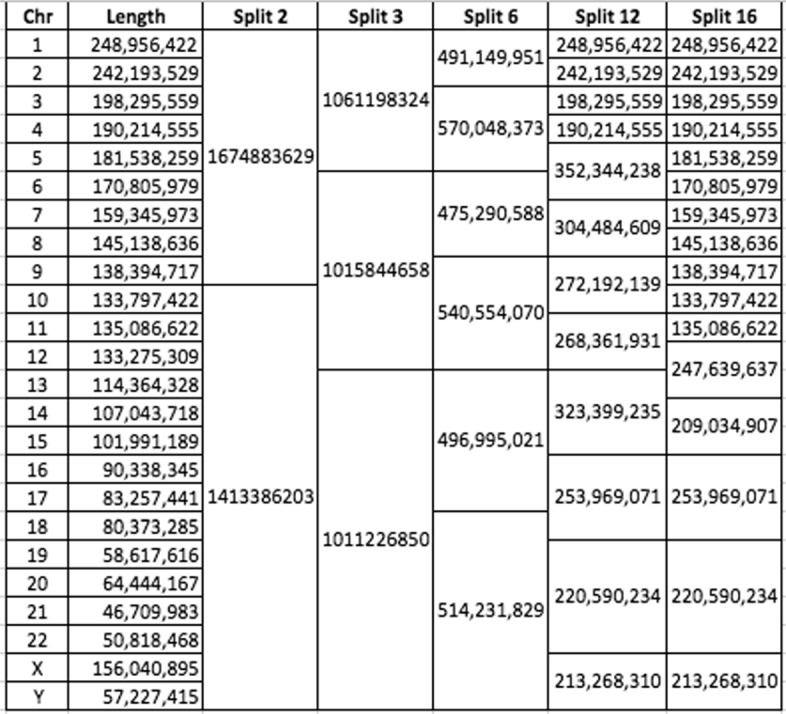
Splitting the genome by chromosomes

Horizontal lines segregate the chunks. Numbers indicate the total number of nucleotides in each resultant chunk of data.

The previously discussed optimizations were applied in these experiments for both GATK3.8 and GATK4. For “within-node splitting,” we strove to optimally fill up our 40-core Skylake nodes by adjusting optimization parameters based on the number of chunks being processed in parallel within the node. For example, in GATK3.8 the optimal thread count for a tool may be around 10 threads, but we set the thread count for each chunk to 3 when the input is split into 12 chunks, while keeping all computations on the same node. Parallel garbage collection degrades the performance of BaseRecalibrator at lower thread counts and was therefore not used in the splitting experiments. Parallel GC was used with MarkDuplicates, but with only 2 threads, as that was optimal.

**GATK3.8 results**

**Fig. 4:**
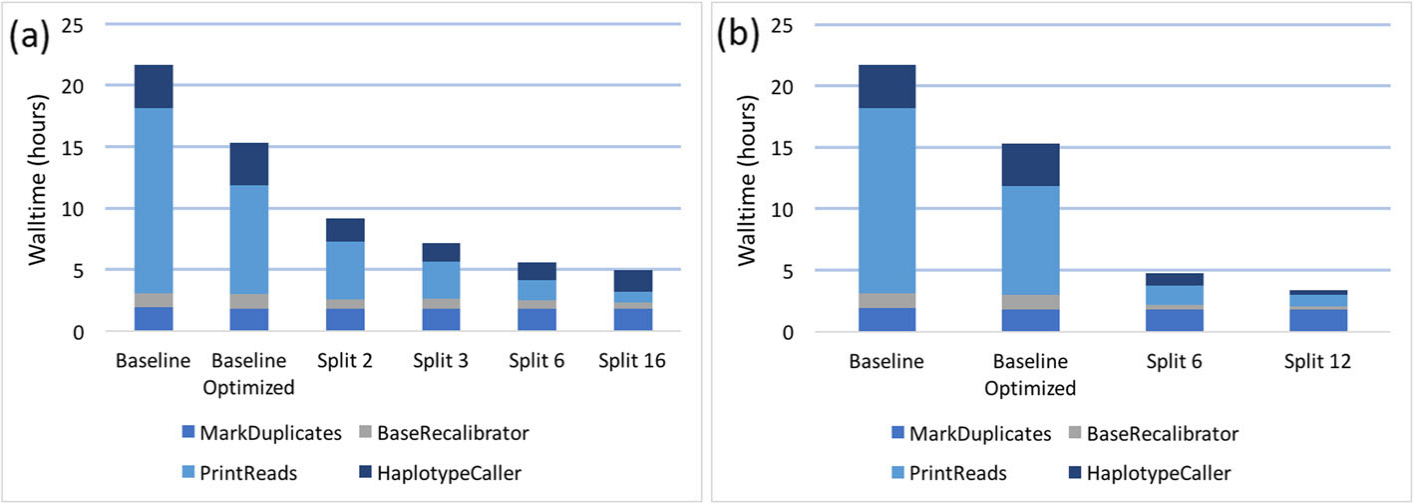
Effects of data-level parallelization in GATK3.8. Sample: NA12878 WGS. The “Baseline” was a naive approach where we gave each tool 40 threads (1 thread per core). The “Baseline Optimized” gave each tool 40 threads, except for PrintReads, which utilized 3 threads. MarkDuplicates and BaseRecalibrator were given 2 and 20 parallel garbage collection threads, respectively. “Split 2,” “Split 3,” etc. means that the aligned sorted BAM was split into 2, 3, etc. chunks, as shown in Table 2. Panel (**a**) shows experiments with chunks computing on the same node. In panel (**b**) computation was spread across nodes in groups of 3 chunks per node

For within-node parallelization beyond three chunks, the benefit of splitting the data begins to be counteracted by the degradation in performance caused by decreasing the thread count of each tool (Fig. 4a). Thus it makes sense to spread execution over multiple nodes. We tested processing 6 chunks on 2 nodes, and 12 chunks on 4 nodes - thus keeping to 3 chunks per node (Fig. 4b). This further reduced the total walltime, although perhaps at a higher compute cost.

**GATK4 results**

**Fig. 5:**
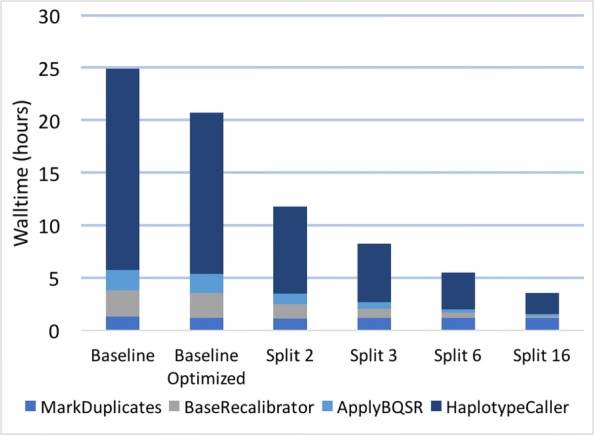
Effects of data-level parallelization in GATK 4. All compute was kept within the same node. Sample: NA12878 WGS. “Split 2,” “Split 3,” etc. means that the aligned sorted BAM was split into 2, 3, etc. chunks, as shown in Table 2

Splitting the aligned sorted BAM into chunks is simple in GATK4, as the only multithreaded tool is HaplotypeCaller. We again split into 2, 3, 6, and 16 chunks, which were kept on the same node, and the PairHMM thread count for HaplotypeCaller was adjusted accordingly (Fig. 5). In contrast to the results we observed for GATK3.8, the walltime keeps improving when splitting all the way down to 16 chunks.

### Throughput

**Fig. 6:**
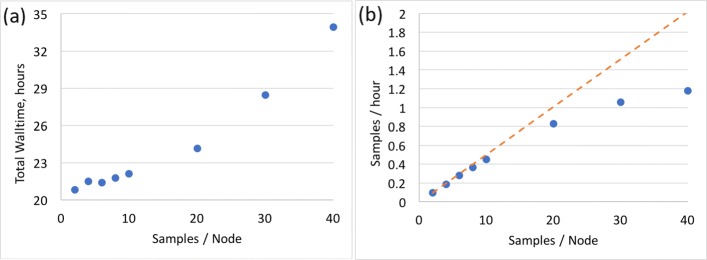
GATK4 throughput testing. Total walltime was benchmarked while running multiple samples simultaneously on the same node. As more samples are placed on the node, the threads given to HaplotypeCaller were reduced accordingly. Sample: NA12878 WGS. **a** Total walltime for running a batch of many samples on the same node. **b** Number of samples effectively processed per hour

When optimizing throughput, one is maximizing the number of samples processed per unit time, albeit at the cost of higher walltime per sample. Because GATK4 is at present single-threaded by design, it lends itself extremely well to this kind of optimization. We created 40 copies of the NA12878 aligned sorted BAM file and processed them in parallel on a single 40-core node (Fig. 6). The overall walltime does increase as one adds more samples to a node, probably due to contention for memory access and possibly disk I/O. However, the overall throughput increases substantially up until around 20 samples per node. Placing more than 20 samples on a 40-core Skylake node is probably not cost-effective.

## Discussion

The tested optimizations intended to speed up computation in individual GATK tools are summarized in Table 3. When applied together, these optimizations significantly reduce the walltime on NA12878 WGS 20X (no splitting by chromosome). In GATK3.8 the MarkDuplicates → BaseRecalibrator → PrintReads → HaplotypeCaller walltime went from 21.7 hours down to 15.3 hours (29.3% improvement). In GATK4 the MarkDuplicates → BaseRecalibrator → ApplyBQSR → HaplotypeCaller walltime went from 24.9 hours to 20.7 hours (16.9% improvement). Note that the walltime is fairly comparable between the two GATK versions despite the single-threaded nature of GATK4, highlighting the performance optimizations introduced into that new release due to complete rewrite of many portions of the code.

**Table 3:** Summary of optimized parameter values

Tool name	GATK3.8		GATK4		
	PGC	Tool threads	PGC	Async	AVX threads
MarkDuplicates	2 threads	1	2 threads	N/A	N/A
BaseRecalibrator	20 threads	-nct 40	20 threads	Yes for Samtools, No for Tribble	N/A
ApplyBQSR	off	-nct 3	off		N/A
HaplotypeCaller	off	-nt 1 -nct 39	off		8

Further walltime improvement can be achieved via splitting the aligned sorted BAM by chromosome. In GATK3.8 the walltime is reduced down to 5 hours when BAM is split into 16 chunks running on the same node – a 76.9% improvement relative to the unoptimized, unsplit configuration. Further benefit can be achieved by splitting into 12 chunks across 4 nodes: down to 3.4 hours (84.3% total improvement). A similar walltime of 3.6 hours is accomplished in GATK4 by splitting into 16 chunks running on the same node – potentially a very cost-effective solution.

To assess the financial costs and benefits resulting from the various configurations of the pipeline, we calculated the dollar amount for our runs based on AWS pricing. All our nodes are built with 40-core Skylake CPUs and 192 GB of RAM. This does not exactly match any of the AWS Skylake instances: c5.9xlarge gives 36 cores and 72 GB of RAM, and c5.18xlarge gives 72 cores and 144 GB of RAM. Our optimizations do aim to maximally pack our nodes with processes, but 72 GB of RAM would probably be insufficient for some high-throughput configurations. Thus Table 4 gives cost estimates for both types of instances, with the understanding that true values are somewhere in between. The Google cloud provides n1-standard-32 instances with 32 cores and 120 GB of RAM, which are more similar to our nodes and therefore provide a closer benchmark. Their cost is $1.51 per hour, which is very close to the AWS c5.9xlarge at $1.52 per hour, and therefore the same dollar estimates apply.

**Table 4:** Financial costs per sample when running an optimized pipeline, based on AWS on-demand pricing as of August 2019: c5.9xlarge at $1.53 per hour and c5.18xlarge at $3.06 per hour

GATK version	Splitting	Samples	Nodes	Walltime, hrs	c5.9xlarge	c5.18xlarge
GATK 4.0.1.2	no splitting	1	1	20.7	$31.7	$63.3
GATK 3.8	no splitting	1	1	15.3	$23.4	$46.8
GATK 3.8	12 chunks	1	4	3.4	$20.8	$41.6
GATK 3.8	6 chunks	1	2	4.7	$14.4	$28.8
GATK 3.8	16 chunks	1	1	5.0	$7.7	$15.3
GATK 4.0.1.2	16 chunks	1	1	3.6	$5.5	$11.0
GATK 4.0.1.2	no splitting	40	1	34.1	$1.3	$2.6

Configurations are sorted by cost.

The data emphasize the trade-off between speed and per-sample cost of the analysis. One could achieve the two types of optimizations outlined in the Background section, using our recommendations as follows. Maximizing speed: to minimize the time to process a single sample, useful in time-critical situations, i.e. when a patient has a critical or rapidly developing condition, use GATK3.8 by splitting the sample into 12 chunks and computing across 4 nodes; resultant walltime is 3.4 hours at the cost of $41.60 on c5.18xlarge. Maximizing throughput: to maximize the number of samples processed per unit time, cost-effective for routine analyses or large population studies, use GATK4.0.1.2 by running 40 samples on one node; total walltime is 34.1 hours with 1.18 samples processed per hour at the cost of $2.60 per sample.

Our study does not encompass the performance issues of Spark code in GATK4, because that functionality was not ready for use as of the time of this writing.

## Conclusions

In this paper, we presented efficient methodology for running the Best Practices variant calling pipeline in a time-sensitive manner by employing run-time optimizing software parameters and data-level parallelizations. We showed a significant improvement in run time on whole human genome data, compared to previous benchmarking efforts. Both GATK3.8 and GATK4 are still useful, for different purposes. The Spark functionality of GATK4 is expected to bring still further speedups to this widely used and valuable code base.

## Data Availability

The sequencing reads for NA12878 were downloaded from Illumina BaseSpace using a process that requires creation of account as described on theirwebsite . The dbSNP build 146 was downloaded from the NCBI FTP site

## Abbreviations

AVX: Advanced vector extensions
AWS: Amazon web services
BQSR: Base quality score recalibration
CPU: Central processing unit
GATK: Genome analysis toolkit
GC: Garbage collection
GKL: Genomics kernel library
HPC: High performance computing
I/O: input-output
PGC: Parallel garbage collector
RAM: Random access memory
SNP: Single nucleotide polymorphism
WES: Whole exome sequencing
WGS: Whole genome sequencing
