# Correction to: Recommendations for performance optimizations when using GATK3.8 and GATK4

**DOI:** 10.1186/s12859-019-3277-4

**Published:** 2019-12-17

**Authors:** Jacob R. Heldenbrand, Saurabh Baheti, Matthew A. Bockol, Travis M. Drucker, Steven N. Hart, Matthew E. Hudson, Ravishankar K. Iyer, Michael T. Kalmbach, Katherine I. Kendig, Eric W. Klee, Nathan R. Mattson, Eric D. Wieben, Mathieu Wiepert, Derek E. Wildman, Liudmila S. Mainzer

**Affiliations:** 1 National Center for Supercomputing Applications, University of Illinois at Urbana-Champaign, 1205 W. Clark St., Urbana, IL USA; 2 Department of Research Services, Mayo Clinic, 200 1st St. SW, Rochester, MN USA; 3 Department of IT Executive Administration, Mayo, Clinic, 200 1st St. SW, Rochester, MN USA; 4 Department of Health Sciences Research, Mayo Clinic, 200 1st St. SW, Rochester, MN USA; 5 Department of Crop Sciences, University of Illinois at Urbana-Champaign, 1102 S. Goodwin Ave., Urbana, IL USA; 6 Department of Electrical and Computer Engineering, University of Illinois at Urbana-Champaign, 306 N. Wright St., Urbana, IL USA; 7 Department of Biochemistry and Molecular Biology, Mayo Clinic, 200 1st St. SW, Rochester, MN USA; 8 Department of Molecular and Integrative Physiology, University of Illinois at Urbana-Champaign, 407 S. Goodwin Ave., Urbana, IL USA; 9 Institute for Genomic Biology, University of Illinois at Urbana-Champaign, 1206 W Gregory Dr., Urbana, IL USA

**Correction to: BMC Bioinformatics (2019) 20: 557**

**https://doi.org/10.1186/s12859-019-3169-7**

Following publication of the original article [1], the author explained that Table 2 is displayed incorrectly. The correct Table 2 is given below. The original article has been corrected.

**Table 2:**
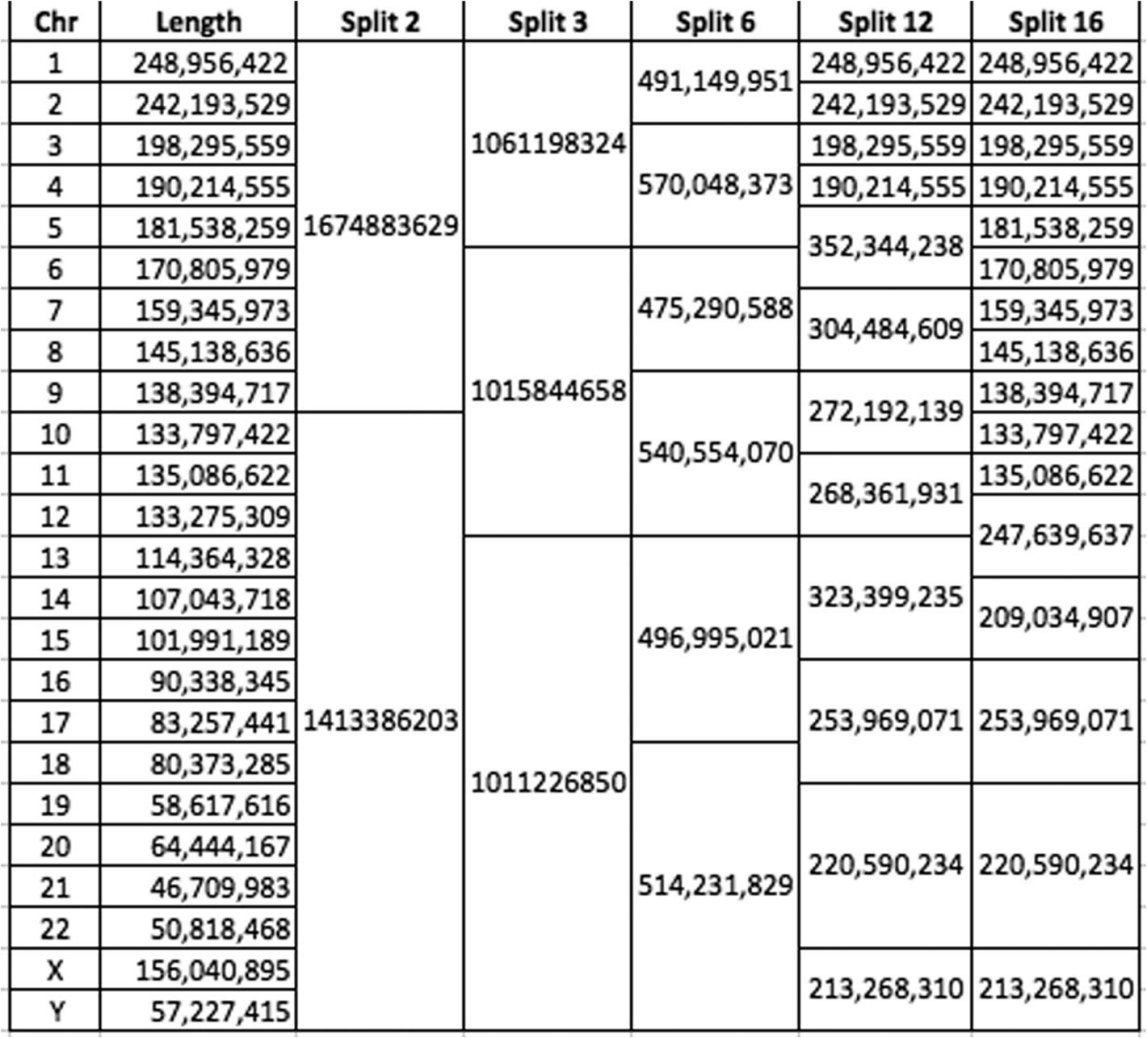
Splitting the genome by chromosomes

Horizontal lines segregate the chunks. Numbers indicate the total number of nucleotides in each resultant chunk of data
